# A Large Sample Survey of Suicide Risk among University Students in China

**DOI:** 10.1186/s12888-021-03480-z

**Published:** 2021-09-28

**Authors:** Ran Wu, Hong Zhu, Zeng-Jian Wang, Chun-Lei Jiang

**Affiliations:** 1 Counseling and Psychological Services Center, East China Normal University, Shanghai, China; 2 Department of Stress Medicine, Faculty of Psychology, Second Military Medical University, Shanghai, China; 3 Center for the Study of Applied Psychology, Key Laboratory of Mental Health and Cognitive Science of Guangdong Province, School of Psychology, South China Normal University, Guangzhou, Guangdong China; 4 Key Laboratory of Brain, Cognition and Education Sciences (South China Normal University), Ministry of Education, Guangzhou, Guangdong China

**Keywords:** suicide ideation, suicide attempt, suicide risk, psychopathology, university students

## Abstract

**Background:**

This study aimed to examine suicide ideation, suicide attempts, and suicide risk by examining a large sample of Chinese university students and identify the predictive factors, including depressive and anxiety symptoms, for suicide attempt and suicide risk.

**Methods:**

We recruited 6,836 students (aged 18–30) based on all students enrolled in 2016 from one university using cluster sampling. They completed four questionnaires: the Beck Scale for Suicide Ideation and the Suicidal Behaviors Questionnaire-Revised were used to measure suicide risk, and students’ depressive/anxiety symptoms were estimated using Patient Health Questionnaire and Generalized Anxiety Disorder Scale.

**Results:**

Four major findings emerged. First, 18% of the students showed high suicide ideation, 14.5% showed suicide risk, 18.8% had suicide plans, and 1% had attempted suicide. Second, a weak sense of life’s value was common among university students, as 61.4% of students considered suicide as a way to end or evade problems. Third, the results of the binary logistic regression showed that education, suicide ideation, including the wish to die, attitude toward suicide, specificity/planning of suicide, and deception or concealment of contemplated suicide were predictive factors of suicide attempt and suicide risk. The variable “deterrents to active attempt” was also a predictive factor of suicide risk. Fourth, depressive and anxiety symptoms did not significantly predict suicide attempts or suicide risk. Only 10.8% and 5.6% of the students had self-reported scores above the clinical cut-off points for depression and anxiety, respectively.

**Conclusions:**

This study highlighted the prevalence of suicide risk among Chinese university students. The high risk of suicide may not only be due to affective disorders, but also a weak sense of life’s value or other reasons. Suicide ideation that significantly predicts suicide risk can be used for suicide risk assessment. Universities should provide appropriate life education and suicide prevention and intervention such as teaching instructors gate-keeper skills.

## Highlights

1. Among Chinese university students, 18% reported high suicide ideation, 14.5% suicide risk, 18.8% suicide plans, and 1% had attempted suicide.
2. 61.4% of the students thought suicide was a way to end or evade problems, reflecting a weak sense of life’s value.
3. Education, suicide ideation including a wish to die, attitude toward suicide, suicide specificity/planning, and deception or concealment of contemplated suicide were predictors of suicide attempts and risk.
4. Depressive and anxiety symptoms did not significantly predict suicide attempts or risk. Only 10.8% and 5.6% of the students at suicide risk exceeded the threshold for depression and anxiety, respectively.

## Background

Suicide is the second leading cause of death worldwide in young people aged 15–29 [1]. Suicide ideation, plans, and attempts have been identified as important precursors of death from suicide [2–4]. A report by the World Health Organization (WHO) showed that the global lifetime prevalence of suicide ideation is 9.2% and that of suicide attempts is 2.7% [1]. Moreover, several studies found that suicide ideation and attempts were more common among Chinese university students than in other countries [5, 6]. For example, a meta-analysis showed that the overall lifetime prevalence of suicide ideation among Chinese university students was 10.7%, higher than the cross-national prevalence among young adults across 17 countries including Africa, the Americas, Asia and the Pacific, Europe and the Middle East (9.2%) [7, 8]. More recent studies found a high incidence rate of suicide ideation (13.2%) and suicide attempts (3.4%) among 730 university students in Jilin province in China [9]. One-third of adolescents with suicide ideation will ultimately to attempt suicide within 12 months [2], and people who attempt suicide have a 12-month risk of suicide of 1.6% [8]. The high lethality is one of the reasons why it is critical to understand suicide ideation.

Existing studies and theories of suicide have explored its associated risks and causes from a different perspective. However, most theories have not been examined among samples of college students and have limited applicability to college students. Many studies with college students have identified factors associated with suicide ideation. For example, factors such as mental disorders, substance abuse, childhood adversity, and identification as sexual minorities may predict suicide ideation [10, 11]. However, few studies have focused on the risk factors for suicide attempts or examined the relationship between suicide ideation and suicide risk among college students.

Suicide not only threatens the life safety of university students, but is also closely associated with their physical and mental health and academic performance [5, 12]. Therefore, the prevalence and characteristics of suicide risk in Chinese university students and the associated risk factors need to be identified to further inform health providers and policy makers. Thus, our study aimed to (1) provide data on suicide ideation, suicide attempt, and suicide risk among university students in China, (2) identify the characteristics of suicide ideation, (3) identify the predictive factors for suicide attempt and suicide risk, and (4) evaluate the relationship between suicide and depressive and anxiety symptoms in university students.

## Methods

### Selection of study participants

This study was conducted in November and December 2016 at a public comprehensive university located in Shanghai, China, a university enrolling approximately 30,000 students from all over China.

**Fig. 1:**
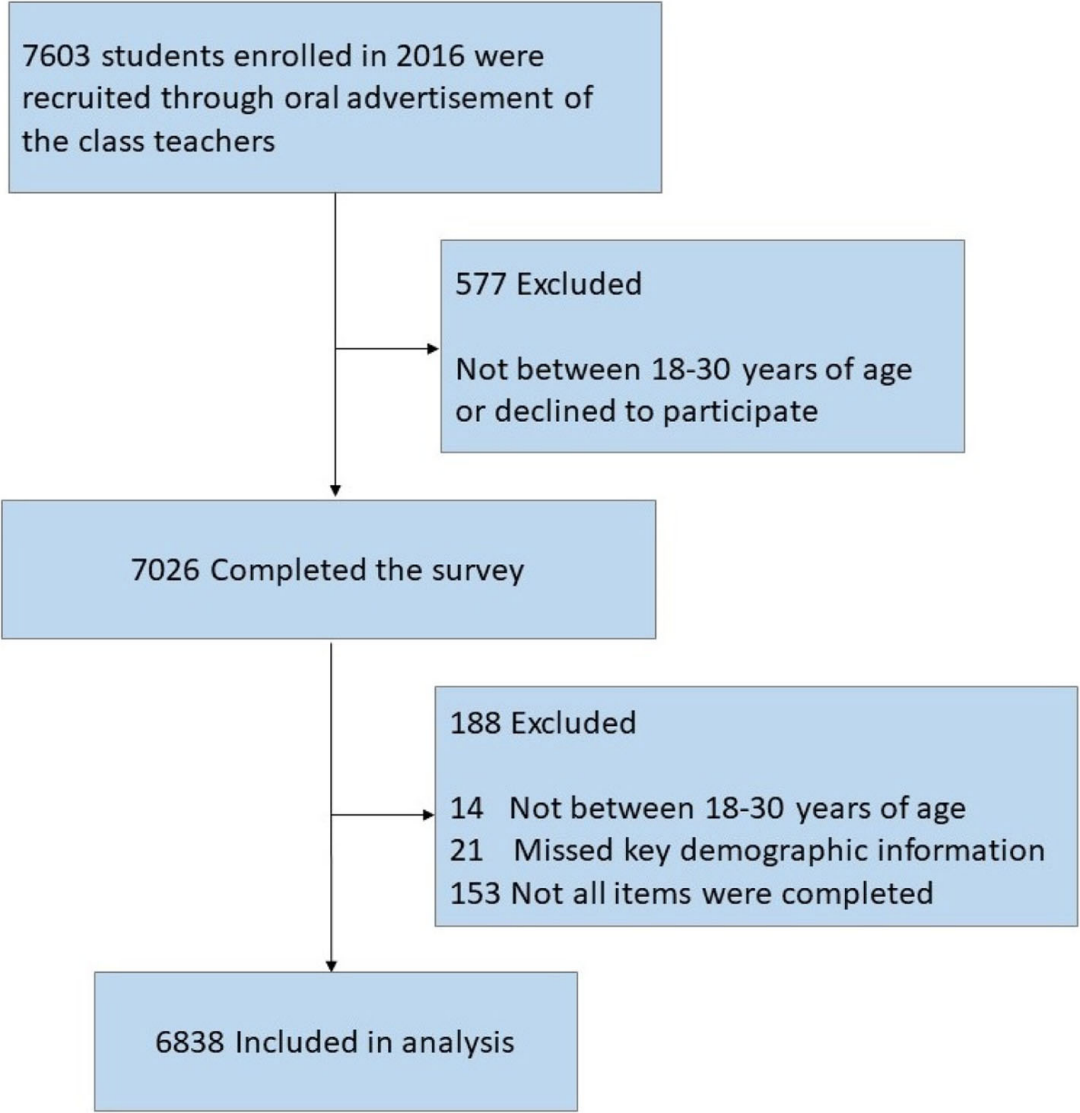
CONSORT Diagram Detailing Study Flow of Participants from Screening to Analysis

Using cluster sampling, all 7603 full-time undergraduate and graduate students enrolled in 2016 were initially recruited through oral advertisement by their class instructors. We excluded 577 participants who: (1) were not between the ages of 18 to 30, (2) declined to participate, (3) did not complete all items, and/or (4) were missing key demographic information. The present study included 6,838 participants; the recruitment flow diagram is shown in Fig. 1.

The students provided informed consent before answering a questionnaire. They were told that their participation was voluntary and that they had the right to refuse and stop the procedure at any time. Ethical approval for the study was obtained from the Committee on Ethics of Biomedicine Research, Second Military Medical University (20170305). The study was conducted in accordance with the relevant guidelines.

### Measures

Suicidality was measured using the Beck Scale for Suicide Ideation (SSI) and Suicidal Behaviors Questionnaire-Revised (SBQ-R), and psychopathology was assessed using the Patient Health Questionnaire (PHQ-9) and the Generalized Anxiety Disorder (GAD-7) scale. Demographic characteristics such as sex, age, education level, and course major were also recorded.

**The SSI** is a self-reported questionnaire that has been widely used to assess current suicide ideation [13]. Suicide ideation means thoughts about death or wanting to be dead [14]. The questionnaire includes 19 items, rated on a 3-point scale ranging from 0 (non-existent) to 2 (obviously existent). Higher total scores represent high suicide ideation. The SSI has demonstrated good reliability and validity [15]. We used the SSI Chinese version [16]. In this study, Cronbach’s alpha coefficient for the SSI was 0.80. We set a total score of SSI ≥ 6 as the cut-off value suggesting high suicide ideation [17]. Question (Q) 12 (i.e., specificity/planning of suicide) and Q17 (i.e., suicide note) were considered essential as they tested the level of specificity of the suicide plan and the presence of a prepared suicide note, respectively [13].

**The SBQ-R** is a brief self-report measure of the frequency and severity of suicidal behaviors and history of suicide attempts [18]. The SBQ-R consists of four items, each tapping a different dimension of suicidality. Higher total scores represent more severe suicidal behaviors. A previous study reported an acceptable Cronbach’s alpha for the SBQ-R [18]. The Chinese version of the SBQ-R was used in this study [19]. The Cronbach’s alpha coefficient in this study was 0.79. A cut-off score of SBQ-R ≥ 7 was used for identifying groups at risk of suicide in this study [18]. We extracted Q1 of the SBQ-R (i.e., past suicide attempts) as the variable indicating attempted suicide or a potentially self-injurious behavior associated with at least some intent to die [14, 18].

**The PHQ-9** is derived from the Primary Care Evaluation of Mental Disorders (PRIME-MD) and has previously been used to assess depressive symptoms based on the two weeks prior to the test [20]. The questionnaire includes nine items rated on a scale ranging from 0 to 3, with the total summed score ranging from 0 to 27. Higher total scores represent more severe depressive symptoms. Scores ranging from 5 to 9 indicate mild depression, 10 to 14 indicate moderate depression, 15 to 19 indicate moderately severe depression, and ≥ 20 indicate severe depression. The Chinese version of the PHQ-9 used in this study has been shown to have good reliability and validity [21, 22], and in this sample, the Cronbach’s alpha was 0.86. A total score of PHQ-9 ≥ 10 was set as the screening threshold for moderate depression and higher [22].

**The GAD-7** is a 7-item self-rating instrument which is used to screen for generalized anxiety disorder and assess its severity [23]. Each item is evaluated by the frequency of symptoms present over the previous two weeks, rated on a scale ranging from 0 (not at all) to 3 (nearly every day). Higher total scores represent more severe anxiety symptoms. Scores of 5 to 9 indicate mild anxiety, 10 to 14 moderate anxiety, and 15 to 19 severe anxiety. The Chinese version of GAD-7 has been widely used and shown to have good reliability and validity [24], and in this study, we adopted a total score of GAD ≥ 10 as the cut-off for significant anxiety problems [24].

### Statistical analyses

Descriptive statistics were used to show the distributions for suicide ideation (SSI) and suicide risk (SBQ-R) scores, including mean, standard deviation, and percentage. Independent *t*-tests were performed to compare differences in suicide ideation and suicide risk across groups by sex, education level, and students’ course majors. Chi-square tests compared differences in proportions across groups by sex, education level, and students’ course majors between high and low risk of suicide ideation and suicide risk. Descriptive data of the 19 SSI items were assessed to clarify suicide ideation in the students.

The enter method of binary logistic regression analyses was used to predict suicide attempt and high suicide risk using demographic factors (i.e., sex, education, and major), suicide ideation (for all SSI items more than 10% of students scored above 0: SSI Q2, Q8, Q10, Q11, Q12, and Q19), and psychopathology (depressive and anxiety symptoms). When used as an independent variable, we assigned “never attempted suicide” (options A–D on SBQ-R Q1) as “0,” and “ had attempted suicide at least once” (options E–F on SBQ-R Q1) as “1.” When suicide risk was used as an independent variable, we assigned “low suicide risk” (SBQ-R < 7) as “0,” and “high suicide risk” (SBQ-R ≤ 7) as “1.” In the sample, 1% (*n* = 66) and 14.5% (*n* = 992) of the participants met criteria for inclusion in the “suicide attempt” group and “high suicide risk” group, respectively. As reported in previous studies, a matching ratio up to 5:1 (control to treatment) was implemented as it is associated with the lowest bias and a higher precision [25, 26]. In this study, the simple random samples of 330 and 4,960 control cases were drawn using the random number generators function in SPSS, respectively.

According to the regression analyses results, Pearson’s correlation analyses examined the relationship between suicidality and depressive and anxiety symptoms. In addition, we examined the depressive and anxiety symptoms that exceeded the selected thresholds for the students with different suicide risks. All data were analyzed utilizing IBM SPSS Version 23 (2015; Armonk, NY: IBM Corp.). A two-tailed significance level was set at *p* < 0.05 for all analyses.

## Results

A total of 6,836 responses were included in the final analytic sample. The average age of the participants (n = 6,836; 35.5% male) was 20.95 years (*SD =* 2.7). The demographic characteristics of the sample are summarized in Table 1.

**Table 1:** Demographic characteristics of the study sample (*N* = 6836)

		*n*	%
Sex	Male	2,428	35.5
	Female	4,408	64.5
Education	Undergraduate	3,175	46.4
	Graduate	3,661	53.6
Major	Liberal arts	2,878	42.1
	Science & Engineering	3,958	57.9

### Distribution of suicide risk

We found that 18% (*n*=1230) of the students had high suicide ideation (SSI ≥ 6), and 14.5% (*n*=991) had suicide risk (SBQ-R ≥ 7). In addition, 18.8% (*n*=1285) had thought about specific plans for suicide (17.2%, *n*=1176) or had specific plans (1.6%, *n*=109; SSI Q12), 1% had attempted suicide (SBQ-R Q1), and 0.4% (*n*=27) had prepared a suicide note (SSI Q17).

### Distribution and characteristics of suicide ideation and behavior

Descriptive data relating to SSI and SBQ-R scores by sex, education level, and major subjects are listed in Table 2. Independent *t*-tests showed that SSI and SBQ-R scores were significantly higher in female, undergraduate, and liberal arts students than male, graduate, and science students, respectively (*p* < 0.05). The proportion of students who had SSI or SBQ-R scores that reached the threshold were also higher in these three groups as determined by Chi-square tests (*p* < 0.05).

**Table 2:** Descriptive data and group difference of scores of SSI and SBQ-R

	SSI							SBQ						
	M(SD)	t-test		SSI Total ≥ 6		Chi-square		M(SD)	t-test		SBQ-R Total ≥ 7		Chi-square	
		*t*	*d*	*n*	%	χ^2^	*d*		*t*	*d*	*n*	%	χ^2^	*d*
Male	3.49 (3.60)	-2.34*	-0.06	401	16.5	5.68*	0.06	4.03 (1.94)	-6.15***	-0.16	287	11.8	21.98***	0.11
Female	3.70 (3.43)			830	18.8			4.36 (2.24)			705	16.0		
Undergraduate	4.32 (4.14)	15.61***	0.38	793	25.0	195.00***	0.34	4.72 (2.52)	17.52***	0.42	665	20.9	197.80***	0.35
Graduate	3.02 (2.67)			438	12.0			3.83 (1.64)			327	8.9		
Liberal arts	3.79 (3.66)	3.42***	0.08	574	19.9	12.63***	0.09	4.36 (2.27)	3.86***	0.01	459	15.9	8.28**	0.07
Science & Engineering	3.50 (3.37)			657	16.6			4.15 (2.04)			533	13.5		
Total	3.62 (3.49)			1231	18.0			4.24 (2.12)			992	14.5		

*p < 0.05, **p < 0.01, ***p < 0.001; SSI, Beck Scale for Suicide Ideation; SBQ-R, Suicidal Behaviors Questionnaire-Revised

### Characteristics of suicide ideation

Table 3 shows descriptive data collected from the 19-item SSI measure. The proportion of partial suicide ideation was above 10%: 18.2% (*n*=1244) of the students indicated a weak (17%, *n*=1162) or medium to strong (1.2%, *n*=82) desire to die (Q2); 14.7% (*n*=1005) reported that they accepted the concept of suicide (Q8); 12.6% reported that they did not have compelling reasons (8.2% had some reason, whereas 4.4% had little or no reason) to abandon suicide (Q10); 61.4% (*n*=4197) thought suicide could help people end or evade problems (Q11); 18.8% (*n*=1285) reported specificity(1.6%) or planning(17.2%) of contemplated attempt (Q12); and 10.7% (*n*=731) of students reported that they would resolutely hide the attempt at suicide (Q19).

**Table 3:** Descriptive data of SSI

	M(SD)	% Suicide Ideation		
		0	1	2
1. Wish to live	1.05 (0.25)	94.8	4.8	0.4
2. Wish to die	1.20 (0.43)	81.7	17.0	1.2
3. Reason for living/dying	1.07 (0.29)	93.6	5.6	0.8
4. Desire to make active suicide attempt	1.10 (0.32)	90.7	8.6	0.7
5. Passive suicidal desire	1.05 (0.25)	96.2	2.9	0.9
6. Time dimension: duration of suicide ideation/wish	1.05 (0.27)	96.1	2.6	1.3
7. Time dimension: frequency of suicide	1.06 (0.25)	94.7	4.8	0.5
8. Attitude toward ideation/wish	1.46 (0.74)	68.5	16.8	14.7
9. Control over suicidal action/acting-out wish	1.07 (0.29)	93.7	5.4	1.0
10. Deterrents to active attempt	1.17 (0.48)	87.3	8.2	4.4
11. Reason for contemplated attempt	2.51 (0.67)	10.2	28.3	61.4
12. Method: specificity or planning of contemplated attempt	1.20 (0.44)	81.1	17.2	1.6
13. Method: availability or opportunity for contemplated attempt	1.12 (0.41)	91.6	5.2	3.2
14. Sense of "capability" to carry out attempt	1.11 (0.36)	90.6	7.9	1.5
15. Expectancy/anticipation of actual attempt	1.07 (0.28)	94.3	4.8	0.9
16. Actual preparation for contemplated attempt	1.01 (0.13)	98.8	1.1	0.2
17. Suicide note	1.03 (0.19)	97.5	2.1	0.4
18. Final acts in anticipation of death	1.04 (0.23)	97.0	2.3	0.7
19. Deception or concealment of contemplated suicide	1.25 (0.63)	85.9	3.3	10.7

SSI, Beck Scale for Suicide Ideation

### Predictive factors for suicide attempt and suicide risk

The results of the binary logistic regressions of factors predicting suicide attempt and high suicide risk are presented in Table 4.

**Table 4:** Binary logistic regression of factors predicting suicide attempt and suicide risk

	Attempted suicide			Suicide risk		
	*Wald*	*P-value*	*OR [95% CI]*	*Wald*	*P-value*	*OR [95% CI]*
**Demographic characteristics**						
Sex	0.55	0.46	1.44 [0.55, 3.83]	0.87	0.35	1.10 [0.90, 1.36]
Education	**5.75**	**0.02**	**0.32 [0.12, 0.81]**	**18.61**	**<0.001**	**0.66 [0.54, 0.79]**
Major	0.16	0.69	1.19 [0.51, 2.78]	0.05	0.83	1.02 [0.84, 1.24]
**Suicide ideation**						
SSI Q2	**5.16**	**0.02**	**2.45 [1.13, 4.92]**	**264.26**	**<0.001**	**4.40 [3.68, 5.26]**
SSI Q8	**14.15**	**<0.001**	**2.56 [1.57, 4.17]**	**207.94**	**<0.001**	**2.29 [2.05, 2.57]**
SSI Q10	0.69	0.41	1.40 [0.63, 3.07]	**33.59**	**<0.001**	**1.61 [1.37, 1.89]**
SSI Q11	0.05	0.82	0.93 [0.50, 1.74]	0.13	0.72	0.97 [0.84, 1.13]
SSI Q12	**22.25**	**<0.001**	**6.40 [2.96, 13.84]**	**411.50**	**<0.001**	**6.13 [5.15, 7.31]**
SSI Q19	**6.11**	**0.01**	**1.87 [1.14, 3.07]**	**71.34**	**<0.001**	**1.67 [1.48, 1.88]**
**Psychopathology**						
PHQ-9	0.23	0.63	0.96 [0.82, 1.12]	0.30	0.59	0.99 [0.96, 1.03]
GAD-7	0.15	0.70	1.03 [0.88, 1.22]	0.23	0.63	0.99 [0.95, 1.03]

OR, Odds Ratio; CI, Confidence Interval; SSI, Beck Scale for Suicide Ideation; PHQ-9, Patient Health Questionnaire; GAD-7, Generalized Anxiety Disorder scale.

The results of the binary logistic regression of factors predicting suicide attempt showed that the resulting model was significant, χ^2^ (345) = 110.92, *p* < 0.001, Nagelkerke *R*^*2*^ = 0.64. The significant measures were found to be education (*OR* = 0.32, 95% *CI* [0.12, 0.81], *p* = 0.02), SSI-2 (wish to die; *OR* = 2.45, 95% CI [1.13, 4.92], *p* = 0.02), SSI-8 (attitude toward suicide; *OR* = 2.56, 95% *CI* [1.57, 4.17], *p* < 0.001), SSI-12 (specificity/planning of suicide; *OR* = 6.40, 95% *CI* [2.96, 13.84], *p* < 0.001), and SSI-19 (deception or concealment of contemplated suicide; *OR* = 1.87, 95% *CI* [1.14, 3.07], *p* = 0.01).

The results of the binary logistic regression of factors predicting high suicide risk showed that the resulting model was significant, χ^2^ (5,952) = 2141.31, *p* < 0.001, Nagelkerke *R*^*2*^ = 0.55. The significant measures were found to be education (*OR* = 0.66, 95% *CI* [0.54, 0.79], *p* < 0.001), SSI Q2 (wish to die; *OR* = 4.40, 95% *CI* [3.68, 5.26], *p* < 0.001), SSI Q8 (attitude toward suicide; *OR* = 2.29, 95% *CI* [2.05, 2.57], *p* < 0.001), SSI Q10 (deterrents to active attempt; *OR* = 1.61, 95% *CI* [1.37, 2.57], *p* < 0.001), SSI Q12 (specificity/planning of suicide; *OR* = 6.13, 95% *CI* [5.15, 7.31], *p* < 0.001), and SSI Q19 (deception or concealment of contemplated suicide; *OR* = 1.67, 95% *CI* [1.48, 1.88], *p* < 0.001).

### Relationship between suicidality and depressive/anxiety symptoms

The correlation between suicidality and depressive/anxiety symptoms showed that suicide ideation and suicidal behavior were positively correlated with depressive and anxiety symptoms, respectively (*p* < 0.001). However, the correlation between scores of the SSI and PHQ-9 (*r* = 0.13), SSI and GAD-7 (*r* = 0.11), SBQ-R and PHQ-9 (*r* = 0.09), and SBQ-R and GAD-7 (*r* = 0.08) were all weak.

Of the students with a history of suicide attempts, 18.2% (*n*=1244) showed elevated depressive symptoms, and 13.6% (*n*=930) showed elevated anxiety symptoms. The proportion of students without a history of suicide attempts showing elevated depressive and anxiety symptoms was 7.6% (*n*=520) and 4.5% (*n*=308), respectively. The proportion of all students with high suicide risk who showed high depressive and anxiety symptoms was 10.8% (*n*=738) and 5.6% (*n*=383), respectively. The proportion of all students with low suicide risk who showed high depressive and anxiety symptoms was 7.2% (*n*=492) and 4.4% (*n*=301), respectively.

## Discussion

The current study is a large sample survey of Chinese university students’ suicidality. The distribution and characteristics of suicide ideation and suicide risk, the predictive factors for suicide attempt and suicide risk, as well as the relationship between suicidality and depressive/anxiety symptoms were measured. This study highlighted the prevalence of suicide risk and its characteristics among Chinese university students.

### Distribution of the students’ suicide risk

In this study, the proportions of students who had high suicide ideation and suicide risk were 18% and 14.5%, respectively. The proportions of the students who had suicide plans, had attempted suicide, and had a suicide note were 18.8%, 1%, and 0.4%, respectively, indicating a high risk of suicide in the future. A meta-analysis found that the proportion of Chinese students who attempted suicide (2.8%) was 2.8 times higher than that observed in our study [27]. Another study also reported a higher proportion of Chinese students who attempted suicide than that observed in this sample [9]. However, the proportions of students with suicide ideation and suicide plans were lower in both studies mentioned above than in this study. A study conducted on US university students found that 24.3% of the students had suicide ideation and 9.3% had attempted suicide [28]; both rates were higher than those in this study. These differences might be due to factors such as geography, time, or different questionnaires.

This study found that sex, majors, and level of education had different effects on suicide risk. The total scores of the SSI and SBQ-R were both significantly higher in female students than male students. The data reported by WHO in 2012 were consistent with our study results, showing that in China, female students had higher suicide rates [1]. The opposite is true in most other countries such as Japan, South Korea, and North America [29]. Therefore, further exploration of the influence of sex differences on suicide risk is necessary.

In addition, this study found that the proportion of undergraduates with suicide risk was significantly greater than that of graduate students, however, it could not be established whether the differences were due to their education level or age. The proportion of liberal arts students with suicide risk was significantly higher than that of science students. Another study also found that the educational level and major could predict suicide ideation among university students [30]. Thus, providing targeted suicide prevention education to students at certain education levels and pursuing certain majors appears to be indicated.

### Characteristics of the students’ suicide risk

This study found that there were some common problems among university students suggesting that they do not cherish life enough and have a weak sense of survival. For example, 18.2% had a desire to die and 14.7% said they accepted the notion of suicide. These results highlight the importance and urgency of strengthening life education. Of all participating students, 61.4% regarded suicide as a way to solve or avoid problems. Some researchers pointed out that the common purpose of suicide is to seek a solution [31]. Suicide can largely be seen as rational and problem-solving behavior by people [32]. Studies showed that the suicide motivation of 70% people was escaping from psychological or physical pain [33]. Tertiary education institutions should guide students to cherish their lives and learn positive problem-solving methods, as well as a positive attitude toward seeking help.

Overall, 12.6% of students reported that they did not have compelling reasons to abandon suicide, which could reflect the lack of social support and lack of hope in life among many students. Universities should be encouraged to help students establish a positive outlook on life and teach them to find hope and resources in difficult situations.

In addition, 10.7% of the participants reported that they would resolutely hide their suicide attempts. In fact, more than half of the students at high risk of suicide (a total of 14.5% of high-risk students) reported that they might hide a suicide attempt, which could increase the difficulty of preventing and intervening in potential suicides. Our results were inconsistent with a previous study, reporting that about half of people who completed suicides communicated their intentions prior to death. One possible reason is that this meta-analysis study included mostly studies from the last century and lacked a sample of Chinese college students. Another possibility is that many students did not realize their desire to communicate suicide during the survey. In general, school authorities must encourage students to seek help and implement a gate-keeper system enabling those who do communicate their wish to attempt suicide to be helped by school authorities.

### Predictive factors of suicide attempt and suicide risk

It was found that education, suicide ideation, including the wish to die, attitude toward suicide, specificity/planning of suicide, and deception or concealment of contemplated suicide were predictive factors of suicide attempt and suicide risk. The variable “deterrents to active attempt” was also predictive factor of suicide risk.

Few studies have compared the suicide risk of undergraduate and graduate students. However, in many universities in China, the same institutions are responsible for providing psychological crisis interventions to both undergraduates and graduate students, and they rarely formulate different policies for them. This study suggested that undergraduates and graduates have significant differences in suicide risk. Thus, targeted suicide interventions should be carried out based on the different needs of different groups. Further research is needed to explore the differences in suicide risk between these two groups in more depth.

A previous study found that people reporting suicidal ideation were likely to attempt suicide within 12 months [3], and about one-third of adolescents with suicidal ideation attempt suicide within a year [2]. Moreover, people who attempted suicide had a 1.6% risk of dying by suicide within a year [4]. It is critical for practitioners and clinicians to remember that a history of suicide ideation can be used as a key indicator in suicide assessment.

In addition, previous studies have suggested that suicide attempts represent a part of a continuous process, originating from the germination of suicide ideation, then formulating suicide plans, and finally died by suicide [34]. This study validated the association of suicide ideation, suicide planning, and attempted suicide. According to the hypothesis of a continuous process, the results of this study could suggest that the beginning of the suicide process involved individuals viewing suicide as a way to solve or escape problems. Subsequently, individuals might develop a desire to escape the problem by death in a difficult situation and begin to make suicide plans. As the specificity of suicide plans increases, they suppress the desire to communicate about suicide, and the probability of individuals taking their own lives increases accordingly. More empirical research is needed to verify this hypothesis.

### Relationship between suicide risk and depression/anxiety

Psychopathology, including depression and anxiety, is almost universally recognized as the most important predictor of suicide [14]. Studies of Chinese college students found that depressive symptoms had direct and indirect effects on suicide risk [30, 35]. However, depression and anxiety scores in this experiment cannot significantly predict suicide attempts or suicide risk. Consistent with previous studies [5, 36], this study found that suicide attempt and students’ suicide risk were positively correlated with depressive/anxiety symptoms. The results suggest that schools need to pay attention to suicide assessment for depressive and anxious students. However, these correlations were weak. Only 18.2% and 13.6% of the students who had attempted suicide exceeded the thresholds used for depression and anxiety, respectively, and 10.8% and 5.6% of the students at suicide risk exceeded the threshold for depression and anxiety, respectively. Thus, most university students with suicide risk might not have affective disorders. Suicide risk might be attributable to weak awareness of cherishing life, poor problem-solving ability, and other reasons. In Chinese society, expressing or defending one’s faith by suicide is highly regarded. For example, some scholars have pointed out that contemporary Chinese college students suffer from “hollow heart disease” or lacking, a sense of value in life, which could increase the suicide ideation and suicide risk of college students. Further rigorous studies are warranted to draw definite conclusions on this issue.

## Conclusion

Overall, our study found a suicide risk in Chinese university students, with the factors influencing the suicide risk including sex, education level, and depressive/anxiety symptoms. Our study also highlighted the fact that engendering a sense of life’s value was crucial to reduce suicide risk among university students from China. More attention must be focused on teaching students to value life.

### Study Limitations

This study had several limitations. First, our study is limited by potential sampling bias, as the sample was from one university; thus, differences between universities could be neglected. Second, because of its retrospective design, the study could not establish a causal relationship between suicide ideation/behavior and attempted suicide. Third, we did not consider other factors that might be associated with suicide risk, such as early life adversity, socio-economic status, and family history of suicide.

## Data Availability

The datasets generated and/or analyzed during the current study are not publicly available due to the relatedness of the recruited data to Tanta university hospitals and the local health authority in the Gharbia governorate but are available from the corresponding author on reasonable request.
